# Cell surface RNA virus nucleocapsid proteins: a viral strategy for immunosuppression?

**DOI:** 10.1038/s44298-024-00051-3

**Published:** 2024-09-02

**Authors:** Alberto Domingo López-Muñoz, Jonathan W. Yewdell

**Affiliations:** Cellular Biology Section, Laboratory of Viral Diseases, NIAID (NIH), Bethesda, MD USA

**Keywords:** Vaccines, Virology

## Abstract

Nucleocapsid protein (N), or nucleoprotein (NP) coats the genome of most RNA viruses, protecting and shielding RNA from cytosolic RNAases and innate immune sensors, and plays a key role in virion biogenesis and viral RNA transcription. Often one of the most highly expressed viral gene products, N induces strong antibody (Ab) and T cell responses. N from different viruses is present on the infected cell surface in copy numbers ranging from tens of thousands to millions per cell, and it can be released to bind to uninfected cells. Surface N is targeted by Abs, which can contribute to viral clearance via Fc-mediated cellular cytotoxicity. Surface N can modulate host immunity by sequestering chemokines (CHKs), extending prior findings that surface N interferes with innate and adaptive immunity. In this review, we consider aspects of surface N cell biology and immunology and describe its potential as a target for anti-viral intervention.

## Introduction

The host immune response drives the evolution of viral immunoevasion mechanisms. Large DNA viruses such as herpesviruses and poxviruses encode the best-known and most obvious immunomodulatory proteins. These include interferon (IFN) antagonists, homologs of host cytokines, CHKs and their receptors, and inhibitors of antigen presentation^1,2^. This diverse arsenal is enabled by a large genome. Indeed, more than 50% of their genome can encode such accessory genes, i.e., genes not required for productive replication^3–5^.

RNA viruses face the same adversaries (e.g., us) but with a much smaller genomic palette (typically 10 to 30 kB) that precludes wholesale capture of host genes for evolutionary remodeling, a favorite trick of large DNA viruses. This puts a premium on multi-tasking both at the level of coding (overlapping genes) and proteins (multifunctionality). Almost all negative and positive strand RNA viruses encode a protein that binds genomic RNA, typically termed N or NP (HIV “*gag*” is an exception). N’s canonical function is binding nascent genomic RNA genome through electrostatic interactions, packing them into long helical ribonucleoprotein complexes and participating in virion assembly. Despite major sequence and structural differences, N proteins from different RNA virus families have been reported to regulate innate and adaptive immunity by suppressing IFN, modulating cytokine production, apoptosis, autophagy, and stress granule formation^6–8^. Thus, N proteins play multiple roles in viral evolution, contributing to viral replication and immune evasion.

**Fig. 1:**
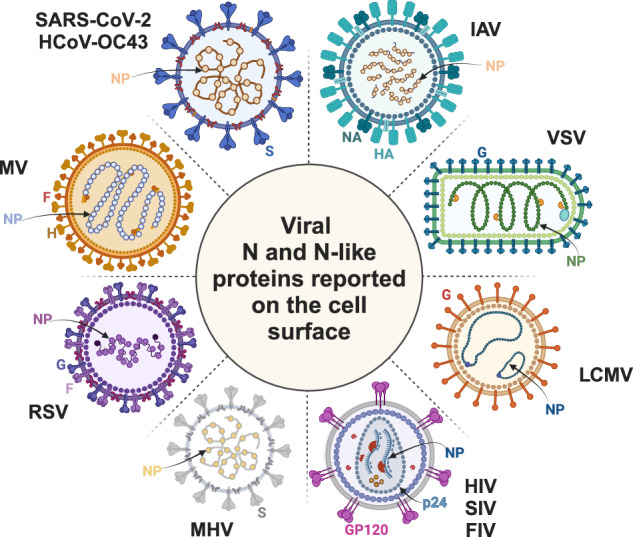
RNA viruses whose N and N-like proteins have been reported on the cell surface during infection. Legend: HA/H (hemagglutinin), NA (neuraminidase), NP (nucleocapsid protein), G (glycoprotein), GP120 (envelope glycoprotein), S (spike), F (fusion protein). The figure was created with Biorender.com.

N proteins lack ER-insertion sequences. Their absence of N-linked glycans added in the endoplasmic reticulum (ER) (though are glycosylated when mistargeted to the ER)^9,10^, confirms their absence from the secretory pathway. Despite this, N protein cell surface expression, detected by antibody (Ab) binding to live cells more than 40 years ago, has proven to be the rule rather than the exception among RNA viruses (Fig. 1, Table 1), including (in order of discovery) influenza A virus (IAV)^11,12^, vesicular stomatitis virus (VSV)^13^, lymphocytic choriomeningitis virus (LCMV)^14,15^, human (HIV), simian (SIV) and feline immunodeficiency virus (FIV)^16–18^, mouse hepatitis coronavirus (MHV)^19,20^, respiratory syncytial virus (RSV)^21^, and measles virus (MV)^22,23^.

**Table 1:** N and N-like RNA-binding proteins from human and animal RNA viruses detected on the surface of infected cells

Virus	Cell type	Cell surface detection (monoclonal (m)Abs unless noted)	Surface host receptor/interacting partners	Biological Activity	References
IAV	D55 (mouse fibroblast) P815 (mouse mastocytoma) MDCK (canine kidney)	Immunofluorescence (IF) with polyclonal (p)Abs IF/Flow Cytometry (FC)/ radioimmunoassay ADCC Hybrid mAb targeted T cell lysis	??	??	^11,12,29–33^
VSV	P815	FC	??	??	^13^
LCMV	MC57G (fibrosarcoma) L929 (mouse fibroblast)	FC Indirect IF and complement-mediated cytolysis	??	??	^14,15^
HIV *gag* p24/17	MT-4 (CD4 T-cell line) persistently infected	IF and radioimmune techniques	??	??	^16,17^
SIV *gag* p24	MT-4 persistently infected	FC	??	??	^18^
FIV	FeL-039 (feline T-cell line)	FC	??	??	^18^
MHV	YAC (mouse fibroblast)	Indirect IF	??	??	^19,20^
RSV	BCH4 (mouse fibroblast), dendritic cells (DCs), and HEp-2 (human carcinoma cell line) persistently infected	FC and IF	T cell receptor (TCR) complex	Inhibits CD4 T cell activation	^21,34^
MV	Human thymic epithelial cells and peripheral blood lymphocytes	FC and IF	FcγRII (CD32) B cell receptor (BCR), unidentified protein receptor	Induces apoptosis. Inhibits cell proliferation, IL-12 secretion, Ig synthesis	^22,23,35,36^
SARS-CoV-2	Several cell lines and human airway epithelium	FC, IF, and ADCC reporter assay	Heparan sulfate, heparin, CC, CXC CHKs	Inhibits CHK-mediated migration, ADCC	^24^
HCoV-OC43	Several cell lines and human airway epithelium	FC, IF, and ADCC reporter assay with pAbs	Heparan sulfate, heparin, CC, CXC CHKs, IL27	Inhibits CHK-mediated migration, ADCC	^25^

Given the typical high anti-N Ab response during infections, surface N is an obvious target of Ab-based adaptive immunity (complement lysis, Ab-dependent cellular cytotoxicity (ADCC) and Ab-dependent cellular phagocytosis (ADCP). Less obvious is surface N manipulation of innate immunity, first reported 20 years ago for MV N as contributing to MV-induced inflammation by inhibiting IL-12 secretion^22,23^. Later, surface RSV N expression was reported to impair CD4 T cell immunological synapse formation^21^. We reported that SARS-CoV-2 N is secreted during infection, binding to the surface of infected cells and non-infected neighboring cells, inhibiting CHK-mediated leukocyte chemotaxis, and enabling activation of Fc-mediated Ab effector functions^24^. Recently, we extended these findings to the human coronavirus (HCoV)-OC43 N protein^25^, suggesting that cell surface N generally contributes to CoV innate immunoevasion.

Large DNA viruses share evolutionary conserved mechanisms to evade immune detection and destruction. One is the secretion of viral proteins that interfere with the cytokine network. These include cytokine homologs, cytokine-receptor homologs, and viral cytokine binding proteins^26–28^. The growing list of surface N proteins (Table 1) suggests RNA viruses might employ an alternative common strategy of using extracellular N to similarly influence innate immunity. Here, we summarize and review current knowledge on surface RNA virus N proteins and their established and potential roles in immunoevasion.

## Summary of studies demonstrating cell surface N expression

Using polyclonal (p)Abs, IAV N was the first N reported to be present on the surface of infected cells^11^ and has been the most intensively studied cell surface N among the different viruses (Table I). Surface N expression was definitively established using monoclonal (m)Abs^12^, a finding confirmed by several laboratories. Passively transferred N pAbs can reduce IAV pathogenesis and IAV replication in mice^32,37,38^. Although anti-N mAbs enable complement-mediated lysis in vitro^12^, in vivo activity of anti-N pAbs is FcγR-mediated and dependent on CD8+ T cells^38^. As anti-N mAbs also mediate ADCP^32^, the extent to which anti-N-based protection is based on Ab interaction with cell surface N (ADCC and complement-mediated lysis) *vs*. N in fragmented virions is uncertain (enhanced phagocytosis leading to increased T cell activation). As with IAV N Abs, passive transfer of LCMV N-specific Abs significantly decreased viral titers in infected mice^15^. The in vivo anti-viral activity of LCMV N-specific mAbs was independent of C3 or FcγR, begging explanation.

HIV, SIV, and FIV encode three structural genes (*gag*, *pol*, and *env*), common to all known replicative retroviruses. Once translated, the *gag* polyprotein is proteolytically divided into four major domains: p17 (matrix), p24 (capsid), p7 (N protein), and p6. Although there are no reports of *gag* p7 (N) cell surface expression, both p17 and p24 have been detected on the surface of persistently HIV-infected cells by immunofluorescence (IF) and radioimmunoassay with mAbs^16,17^. These authors later extended these findings to SIV and FIV *gag* p24 using mAbs^18^, consistent with *gag* cell surface expression being a feature of lentivirus infection.

MHV N protein was detected on the surface of infected cells using IF with mAbs as well as mAb-mediated complement lysis of infected cells. Adoptively transferred mAbs protected mice against lethal MHC infection^19,20^.

Additional biological activities of cell surface N from IAV, VSV, LCMV, HIV, SIV, FIV, and MHV remain to be discovered.

## Cell surface N-mediated immunosuppression

### RSV

RSV N is expressed on the surface of infected cells, including mouse DCs, detected with mAbs by flow cytometry (FC) and IF 24 h post-infection (hpi)^21^. N is detected as early as 1 hpi with either infectious or inactivated virus, demonstrating that surface N derives from the inoculum and not endogenously synthesized protein. By 24 h post-infection, endogenously synthesized N increases the N surface signal. N is released by infected cells, possibly due to secretion by the classical ER to Golgi complex (GC) pathway, but the evidence for this conclusion is limited to marginal co-colocalization with the GC by IF and partial effects of brefeldin A secretion blockade. Soluble recombinant N binds cells, consistent with released N binding accounting for N cell surface expression.

Adding soluble N to DCs or artificial MHC class II bearing membranes impairs their ability to present peptides to naïve CD4 T cells. N did not colocalize with MHC-loaded peptides on artificial membranes but colocalized with TCRs and even induced TCR clustering on T cells, suggesting its interaction with one or more components of the TCR micro cluster complex on the T cell surface, which contains CD2, CD3, CD4, CD28 in addition to the TC. Whether RSV N can also inhibit the activation of CD8 T cells remains unexplored. The relevance of N interference with T cells in vivo remains to be established. This will be difficult, particularly since RSV infection of human CD4 and CD8 T cells^39^ likely contributes to RSV-associated defects in T cell responses.

### MV

The immunosuppressive properties of MV N were discovered by adding recombinant N to mouse and human B cells. This revealed N binding to FcγRII on the surface of B cells, as shown by 90% inhibition using anti-FcγRII mAbs and the ability of FcγRII gene expression to confer N binding to FcγRII negative cells. N binding to B cells reduced immunoglobulin synthesis of activated human B lymphocytes by 50%^35,36^.

Extending these findings, MV N expressed by human thymic epithelial cells and peripheral blood lymphocytes infected with wild-type or vaccine strains was detected on the cell surface with mAbs by FC and IF^22,23^. Newly synthesized N enters the late endocytic compartment via an unknown mechanism. N remains in endosomes if cells lack FcγRII (e.g., T cells). If FcγRII is present, it associates with N and delivers N to the plasma membrane, where it can dissociate and bind FcγRII on non-infected neighboring cells by cell-to-cell contact and cell-free diffusion. N cell surface expression is independent of other viral genes, as it is observed in FcγRII positive cells expressing N from a transgene.

Biologically active N can also be released from dead and dying MV-infected cells and bind other cell surface proteins expressed by human, monkey, and mouse cells. Binding to human T cells requires T cell activation and blocks further proliferation^22^. Binding of N to human thymic epithelial cells induces calcium influx and causes G0/G1 cell cycle arrest^22^. Both cell-derived and recombinant N inhibit IL-12 secretion by human and mouse macrophages. Injecting N or cells expressing a transgene encoding N inhibits mouse ear swelling in an IL-12-dependent allergen model^23^. MV N also binds to the B cell receptor, i.e., cell surface immunoglobulin, inhibiting immunoglobulin synthesis^35,36^.

As with N from other viruses, gauging the in vivo importance of N-based immunosuppression is complicated by the many other effects induced by other viral proteins^40^.

### HCoV

We found that SARS-CoV-2 N is localized on the surface of SARS-CoV-2 infected and transiently transfected Vero, BHK-21, Caco-2, Calu-3, CHO-K1, HEK293-FT cells, with mAbs by IF, FC and ADCC reporter assays^24^. Surface N, as expected, is a target for ADCC^24,25,41^. More recently, we reported that N from the common cold HCoV-OC43 is robustly expressed on the surface of infected cell lines by the same criteria^25^. Pooled human airway epithelial cell cultures infected with SARS-CoV-2 or HCoV-OC43 demonstrated significant levels of cell surface N after 72 hpi by FC with mAbs, showing the relevance of surface N expression to conditions approximating human airway infections. As natural N is not glycosylated (unlike artificially ER-targeted N), surface expression does not entail classical ER to GC export.

We detected surface N on both infected cells and non-infected neighboring cells^24^. N, like all N proteins, is highly positively charged, and binding of endogenous N and cell-derived or recombinant N to cells requires heparan sulfate/heparin (highly negatively charged proteoglycan), as shown by the abrogation of binding by enzymatic or genetic removal of heparan sulfate/heparin. Consistent with this finding, N binds to heparin/heparin sulfate with nanomolar affinity but no other sulfated glycosaminoglycans, and cell binding is blocked by polybrene, a cationic polymer that neutralizes cell surface electrostatic charge^24,25^. N produced by SARS-CoV-2-infected cells is transferred through 3 μm filters to non-infected cells, demonstrating that cell contact is unnecessary. Levels are much higher, however, in co-cultured cells, consistent with parallel and likely more robust transfer by cell contact.

The presence of N in serum within the first few weeks of SARS-CoV-2 infection suggests the physiological relevance of released N^42–44^. The extent to which N detected in these assays is free *vs*. present in ribonucleoproteins, virions, or exosomes remains to be determined^45^. Given the ubiquitous expression of heparan sulfate/heparin on cells, including endothelial cells, it seems unlikely that sufficient N is released by infected cells to saturate available cell surfaces. In extending these findings, Wu et al.^46^ reported that N derived from the Omicron variant binds more weakly to the plasma membrane. They identified STEAP2, a likely non-glycosylated cell surface protein, as a co-receptor in the cell lines tested. RNASeq, however, indicates that STEAP2 mRNA is present at low levels in all human tissues except prostate, inconsistent with STEAP2 being a normal N receptor. In any event, transiently expressed N was reported to mediate RNA and DNA transport to recipient neighboring cells through STEAP2-mediated endocytosis, achieving gene expression in the recipient cells, suggesting another function for N^46^.

Among all SARS-CoV-2 structural (spike, membrane, envelope and N) and accessory proteins (ORFs 3a, 3b, 6, 7a, 7b, 8, 9b, 9c, and 10) screened for interaction against 64 human cytokines by bio-layer interferometry, only N bound 11 CHKs (CCL5, CCL11, CCL21, CCL26, CCL28, CXCL4, CXCL9, CXCL10, CXCL11, CXCL12β, and CXCL14) with micromolar to nanomolar affinity^24^. HCoV-OC43 N binds with high affinity to the same set of 11 CHKs as SARS-CoV-2 N, but also to an exclusive set of 6 additional cytokines (CCL13, CCL20, CCL25, CXCL12α, CXCL13, and IL27)^25^.

*In silico* modeling of interaction with HADDOCK and AlphaFold2-Multimer software between SARS-CoV-2 N and CXCL12β reveals a high specificity of docking^47^. SARS-CoV-2 and HCoV-OC43 N proteins inhibited in vitro CXCL12β-mediated leukocyte migration in chemotaxis assays. Exogenous recombinant N from highly pathogenic (SARS-CoV, MERS-CoV) and common cold HCoV (HKU1, NL63, and 229E) also inhibited in vitro CXCL12β-mediated leukocyte migration. Notably, despite this conserved function, the sequence homology between HCoV N proteins can be considerably low even within the same viral genus (38% between SARS-CoV-2 and HCoV-OC43)^48,49^.

Given the large number of CHKs bound by HCoV N, it will be difficult to gauge their impact in animal models by targeted CHK gene knockout or Ab-mediated interference.

## Concluding remarks

N is typically among the most abundant viral proteins expressed during RNA virus infection. Based on the increasing evidence, N expression on the surface of RNA virus-infected cells is likely to be the rule rather than the exception. There is limited evidence supporting in vivo N surface expression. SARS-CoV-2 N has been detected in lung, intestine, and kidney biopsies from fatal and recovered COVID-19 patients without signs of viral replication^50–52^, consistent with its presence on the cell surfaces. Further, high levels of free SARS-CoV-2 N in the blood and urine of patients correlates with severe disease^53–55^. In vivo N cell surface expression is a critical question for future studies. There is no evidence that N reaches the cell surface via the standard ER to GC secretory pathway; the evidence suggests that N is secreted through a non-canonical secretory pathway^56^, like HIV-Tat protein^57,58^. Several cellular proteins non-canonically exported to the cell surface (e.g., FGF2, tau) bind proteoglycans such as heparan sulfate, which have been shown to mediate the secretion of these proteins to the extracellular compartment^59,60^. This is an obvious starting point for studying the secretion of HCoV N, given its binding to heparin/heparin sulfate. More generally, N protein membrane penetration may be typical of proteins with highly positively charged domains. Cationic proteins (e.g., Tat) penetrate cells and can confer cell penetration when appended to proteins. Anti-DNA Abs have long been known to penetrate living cells and traffic to the nucleus^61^, a charge-dependent process requiring a cationic Ab antigen binding site and cell surface proteoglycans^62^.

Given their common binding to RNA via positively charged domains, it is likely that many, if not all, or nearly all viral N proteins will, like the HCoV N proteins studied, bind to cell surface proteoglycans. Other secreted viral proteins also bind to the cell surface of infected or adjacent cells through proteoglycans. These include innate immune immunosuppressive factors such as herpes simplex virus 2 glycoprotein gG^63^, myxoma virus T1 protein^64^, ectromelia virus E163 protein^65^, vaccinia virus B18 protein^66^, and molluscum contagiosum virus MC54L protein^67^.

N proteins are highly immunogenic, inducing rapid and robust IgG response. IgG Abs against IAV N protein promote viral clearance in mice by mechanisms involving both Fc receptors and CD8 + T lymphocytes^38^, consistent with a contribution from ADCC of viral infected cells and possibly Ab-enhanced DCs cross-presentation of N containing viral debris to activate CD8 + T cells. Anti-N Abs have been shown to improve control of SARS-CoV-2 in mice and hamsters^68–70^. We and others reported HCoVs N as a target for Fc-mediated Ab effector functions, since anti-N Abs trigger infected cell activation of NK cell^24,25,41^.

The strong immunogenicity and antigenic stability of N make it an attractive candidate for vaccines aiming for broad coverage against closely related viruses. A combination of spike+N mRNA (ancestral SARS-CoV-2 sequence, Wuhan-Hu-1) vaccination induced more robust control of the SARS-CoV-2 Delta and Omicron variants in the lungs than spike mRNA alone, and reduced viral load in the upper respiratory tract in preclinical models^70^. An N-based vaccine against IAV elicited significant humoral and cellular NP-specific immune responses and reported to provide an 84% level of protection against PCR-confirmed symptomatic influenza compared to placebo in a phase 2 clinical trial^71^. Similar results have been reported for a SARS-CoV-2 N-based vaccine in hamsters, generating strong and broad-spectrum N immune responses across multiple SARS-CoV-2 variants^72^.

While the most obvious benefit of N-based vaccines is the induction of CD8+ and CD4 + T cell responses, it will be important to assess the contribution of anti-N Abs to viral clearance and protection. As with all human virus protection studies, this will not be an easy task, as the contribution of even CD8 + T cells to protection against acute viral infections remains to be firmly established. It will be equally difficult to establish the role of N proteins in modulating anti-viral immunity, though clues may be offered, ironically, in characterizing human immune responses to N *vs*. viral-receptor-protein-based vaccines by analyzing serum and cell immune signatures. Other clues to the evolutionary importance of N CHK-binding may come from mutational studies that identify residues critical for binding, enabling experiments to determine the fitness of such mutants in animals with various immune defects and resulting evolutionary changes in the mutants.

Although surface N protein expression was discovered nearly 50 years ago, research has been highly sporadic, with only a few dozen studies reported to date. Hopefully, the intense worldwide interest to better understand HCoV immunity, in particular, and viral immunity, in general, will fuel interest in the role of N proteins in viral immunity and immune evasion, leading to developing N based vaccines and possibly even therapeutics.
